# Combinatorial active contour bilateral filter for ultrasound image segmentation

**DOI:** 10.1117/1.JMI.7.5.057003

**Published:** 2020-10-27

**Authors:** Anan Nugroho, Risanuri Hidayat, Hanung A. Nugroho, Johan Debayle

**Affiliations:** a Universitas Gadjah Mada, Department of Electrical and Information Engineering, Yogyakarta, Indonesia; b Universitas Negeri Semarang, Department of Electrical Engineering, Semarang, Indonesia; c MINES Saint-Étienne, SPIN/LGF CNRS UMR 5307, Saint-Étienne Cedex 2, France

**Keywords:** computer-aided diagnosis, radiology, ultrasound, speckle, active contour, bilateral filter

## Abstract

**Purpose:** Utilization of computer-aided diagnosis (CAD) on radiological ultrasound (US) imaging has increased tremendously. The prominent CAD applications are found in breast and thyroid cancer investigation. To make appropriate clinical recommendations, it is important to accurately segment the cancerous object called a lesion. Segmentation is a crucial step but undoubtedly a challenging problem due to various perturbations, e.g., speckle noise, intensity inhomogeneity, and low contrast.

**Approach:** We present a combinatorial framework for US image segmentation using a bilateral filter (BF) and hybrid region-edge-based active contour (AC) model. The BF is adopted to smooth images while preserving edges. Then the hybrid model of region and edge-based AC is applied along the scales in a global-to-local manner to capture the lesion areas. The framework was tested in segmenting 258 US images of breast and thyroid, which were validated by manual ground truths.

**Results:** The proposed framework is accessed quantitatively based on the overlapping values of the Dice coefficient, which reaches 90.05±5.81%. The evaluation with and without the BF shows that the enhancement procedure improves the framework well.

**Conclusions:** The high performance of the proposed method in our experimental results indicates its potential for practical implementations in CAD radiological US systems.

## 1 Introduction

Imaging modalities play a critical role in radiological procedures as they strive to achieve early disease detection. Among the existing modalities, ultrasound (US) is mostly utilized to investigate abnormalities of the glands such as in breast and thyroid screening.^1^ US is real time, low cost, non-invasive, and user friendly.^2^ The gold standard report of Breast Imaging Reporting and Data System (BIRADS)^3^ or Thyroid Imaging Reporting and Data System (TIRADS)^4^ is commonly used by radiologists to analyze US images. These quality assurances consist of valuable points describing shape, margin, orientation, and textural echo of suspicious lesions or nodules. It is worth noting that US visual analysis is expert dependent, which leads to high variabilities in interpretation and clinical recommendations.^5^ Therefore computer-aided diagnosis (CAD) systems have become a second-opinion reader by implementing appropriate image processing algorithms to improve radiologists’ diagnoses. All BIRADS and TIRADS features can be extracted properly only after the US objects are correctly segmented.^6^ Thus, the right segmentation method is a crucial part of accurate diagnosis in CAD systems.

Referring to the latest scientific reviews,^6^^–^^9^ each US segmentation approach has its own pros and cons, but accuracy, automation, and adaptability are the most important in creating an advanced CAD system.^8^^,^^9^ Among the existing methods, active contour (AC) models have been the preferred and most widely used methods due to many advances.^10^^–^^13^ They are self adapting to various object shapes,^13^ have high potential for automatic^11^^,^^12^ and adaptive adjustment,^14^ and can be flexibly integrated into any framework.^15^ Other methods can be ruled out considering some disadvantages such as requiring a lot of intervention, inevitable manual tuning, and strict parameter control. In fact, learning-based methods—even the most advanced ones—depend on the availability of datasets to get their best models. Fortunately, this is not a serious obstacle in developing AC-based segmentation techniques. Edge-based geodesic active contour (GAC)^16^ and region-based Chan–Vese (CV)^17^ are two fundamental AC models. The GAC model employs the local edge information to stop contour evolution so that it is possible to segment the specific lesion among the heterogeneous objects.^18^ However, as the GAC model relies on the edge-function which depends on the image gradient, this model can detect only objects with edges defined by strong gradients.^19^ In fact, if the radiological US images are blurry, then the stopping function is never zero on the edges and the leakage problem may occur through the lesion boundaries. Moreover, GAC is sensitive to the determination of the initial contour. By contrast, the CV model is insensitive to the initialization and independent of the image gradient.^19^ The CV stopping term is derived from the Mumford–Shah energy functional^20^ based on global region information. In this way, the initial contour can be anywhere and the model can quickly detect contours both with or without gradient even for objects with discontinuous boundaries. It should also be noted that CV always considers images as two homogeneous regions, which makes this model fail when dealing with inhomogeneous situations. In cases in which the targeted objects cannot be easily distinguished by global statistic terms, region-based CV may lead to erroneous segmentations.^18^ The problem with this CV model further limits its application in US images as radiological cancerous objects tend to have heterogeneous intensities. GACV^21^ and its modifications^14^^,^^22^ are combined models that seek to integrate advantages of local edge-based GAC and global region-based CV. Unfortunately, as long as noisy US images are not enhanced well, inhomogeneity is still a problem and the generated edge-stopping function remains weak, so the failure of AC evolution continues. Speckle is a major factor that limits the contrast and homogeneity in US imaging, thereby reducing the effective application of image segmentation algorithms.^23^ More specifically, inhomogeneity and weak edges are the basic factors causing improper AC evolution. It may be that the evolution is stuck in the false area (false positive and false negative) or falls into the leakage problem. Hence, integrating local and global information in the AC model supported by an appropriate speckle reduction technique is a significant consideration to obtain accurate US segmentation.

Many efforts have been made in US image enhancement, and a bilateral filter (BF) is effective for speckle reduction compared with other techniques.^23^^–^^25^ The BF was introduced by Tomasi and Maduchi^26^ in which every value of output pixel in the whole image is a weighted Gaussian average of its neighbors in both intensity and spatial range. Based on our preliminary studies,^27^^,^^28^ the BF can effectively increase homogeneity, enhance object boundary, and preserve useful information in the US images. Moreover, the newest modified BF is fast and robust at large noise levels, thus making it work better and have more power.^29^ In this paper, a combinatorial framework of a region-edge based hybrid AC model with a BF enhancement called active contour bilateral filter (ACBF) is proposed for US image segmentation. The level set approach^30^ is adopted to represent hybrid AC evolution due to its superiority in handling topology changes such as merging and splitting for multiple object segmentation. Avoiding troublesome operator intervention, a simplified CV composition is introduced in this proposed hybrid AC. Employment of the BF is the reliable first stage to suppressing speckle and increasing pixel homogeneity so that the global statistical term in the simplified CV works effectively. A binary stopping function (BSF) is then generated through the zero-level set of the simplified CV after it converges. The robustness of the BSF provides certainty that local evolution of GAC is prevented from leaking and is kept away from being trapped into local minimal. A convergence criterion based on tolerable error area and contour length is also embedded so that the switching evolution from global to local takes place adaptively. In this way, the ACBF optimally combines the advantages of the BF, GAC, and CV. Furthermore, the reaction diffusion (RD) concept is applied to maintain stability of the level set evolution as it is simple, practical, and provably more effective than other regularization according to an experimental study by Zhang et al.^31^ And to demonstrate the performance of the proposed method, the ACBF was tested to segment 258 US breast and thyroid images. It is also compared with several AC models and evaluated quantitatively by the Dice coefficient (DC) metric.

The rest of this paper is structured as follows. In Sec. 2, we first review the fundamental concepts of GAC, CV simplification, and BF followed by an explanation of the proposed ACBF. Results and discussion are described in Sec. 3, including the advantages of the proposed ACBF over other models. Comparison of the segmentation performance with and without the BF is also clearly discussed in this section. Then, our work is concluded in Sec. 4.

## 2 Methods

### 2.1 Edge-Based GAC Model

**Fig. 1:**
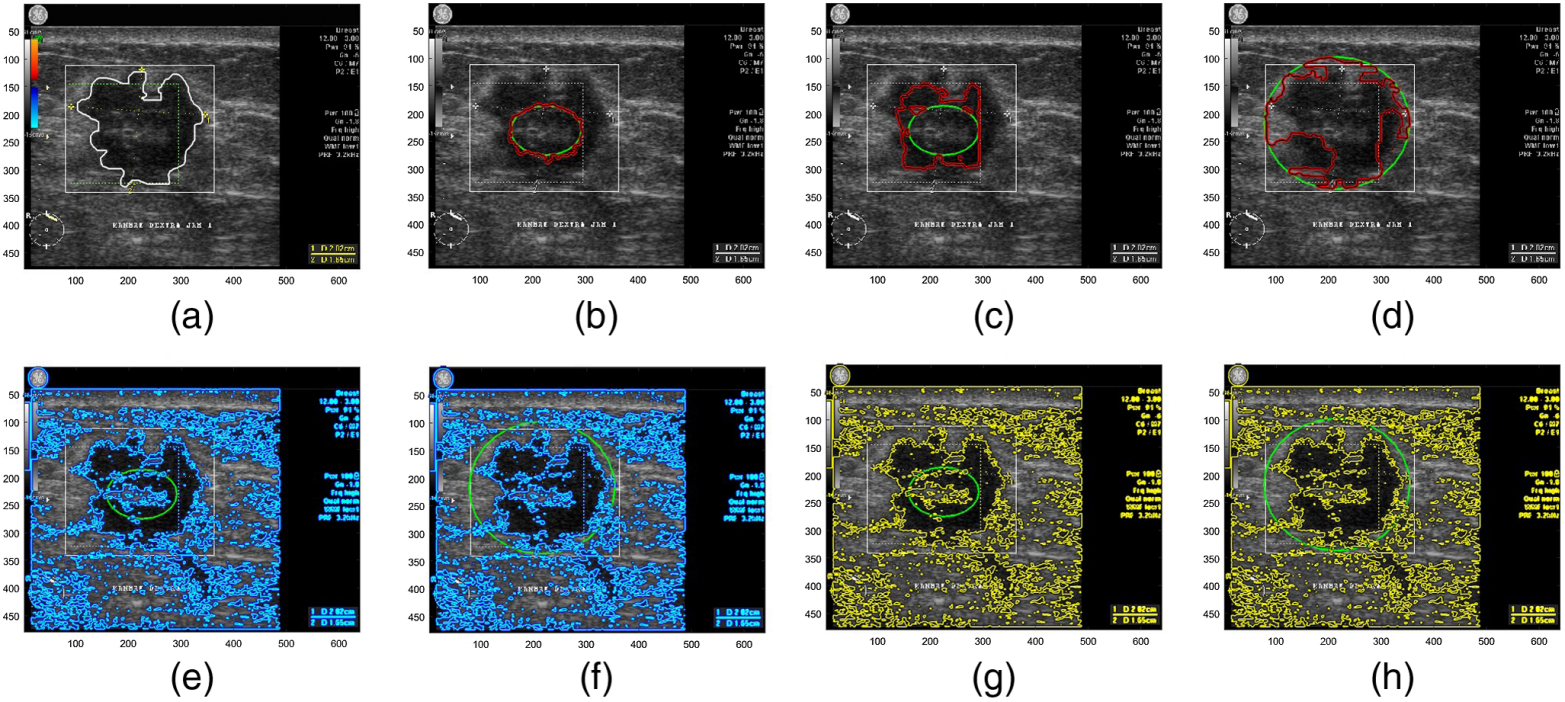
(a). Ground truth. (b)–(d) GAC with (G=3×3; σ=1; v=−1), (G=5×5; σ=3; v=−1), and (G=7×7; σ=5; v=1), respectively. (e) and (f) CV with (μ=0.9, $\lambda_{1}=0.8$, and $\lambda_{2}=0.85$ and $\left(\mu =\lambda_{1}=\lambda_{2}=1\right)$, respectively. (g) and (h) Simplified CV.

Implementation of the level set-based AC model for image segmentation is implicitly expressed by the Lipschitz function as

$$\phi (x,y,t)\{\begin{array}{ll} <0 & \mathrm{for}\;(x,y)\in \Omega^{-}\\ =0 & \mathrm{for}\;(x,y)\in \Phi \\ >0 & \mathrm{for}\;(x,y)\in \Omega^{+} \end{array},$$ (1)

where Φ is a closed contour dividing the image domain Ω, $\Omega^{-}$ describes the area inside Φ, and $\Omega^{+}$ describes the area outside Φ. The dynamic level set function ϕ evolves following time t. Segmentation is expressed by the zero-level set (ϕ=0) at t>0. The GAC model is a local segmentation method constructed by elastic curvature motion $div(\frac{\nabla \phi }{\left|\nabla \phi \right|})$, constant speed movement v, and the edge stopping function (ESF) g. The level set evolution of GAC is formulated as follows:

$$\frac{\partial \phi }{\partial t}=[\mathrm{div}(g\frac{\nabla \phi }{\left|\nabla \phi \right|})+vg]|\nabla \phi |,$$ (2)

where ∇ is the geometric gradient operator and |∇ϕ| drives the normal direction for contour deformation. The initial level set $\left(\phi_{0}\right)$ at t=0 is given as

$$\phi_{0}(x,y)\{\begin{array}{ll} -1 & \mathrm{if}\;(x,y)\in C_{0}\\ +1 & \text{otherwise} \end{array},$$ (3)

where $C_{0}$ is any kind of closed region in domain Ω. The constant v is an adjustable control deformation depending on whether the GAC shrinking (v>0) or expanding (v<0). The conventional g is originally given as

$$g=\frac{1}{1+{\left|\nabla (G_{\sigma }*I)\right|}^{2}},$$ (4)

where (*) is the smoothing convolution operation of image I using Gaussian kernel G with deviation σ. The local evolution ability of GAC is indeed advantageous for segmenting specific lesions. However, generating a powerful ESF through Eq. (4) is hard to achieve. If the discrete gradients of US images are bounded, then ESF g is rarely zero on the object boundaries. So, manually tuning σ is a time-wasting task as shown in Figs. 1(b)–1(d). Two undesired conditions are always prone to occur, i.e., the leakage problem and being trapped into local minimal.

### 2.2 Simplified CV Model as Binary Stopping Function

CV is a global segmentation method driven by updating the average value of pixel intensities inside ($c_{1}$) and outside ($c_{2}$) the contour.

$$\frac{\partial \phi }{\partial t}=[\mu .\mathrm{div}(\frac{\nabla \phi }{\left|\nabla \phi \right|})-\lambda_{1}{\left(I-c_{1}\right)}^{2}+\lambda_{2}{\left(I-c_{2}\right)}^{2}]\delta (\phi ),$$ (5)

where $\lambda_{1},\lambda_{1}$, and μ are the adjustable constants. As proposed by CV,^17^ variational Dirac δ(ϕ) is any function that follows the following:

$$\delta (\phi )=\frac{d}{d\phi }H(\phi )\;\text{and}\;H(\phi )=\{\begin{array}{cc} 1 & \text{if}\;\phi <0\\ 0 & \text{if}\;\phi >0 \end{array},$$ (6)

where H(ϕ) is called the Heaviside function. Considering Eqs. (1) and (6), updated $c_{1}$ and $c_{2}$ in practice are obtained as

$$c_{1}(\phi )=\frac{\int_{\Omega }I(x,y).H(\phi )dx\;dy}{\int_{\Omega }H(\phi )dx\;dy}\;\text{and}\;c_{2}(\phi )=\frac{\int_{\Omega }I(x,y).[1-H(\phi )]dx\;dy}{\int_{\Omega }[1-H(\phi )]dx\;dy}.$$ (7)

By eliminating g and substituting v with region intensity approximation as the fitting term, initialization $C_{0}$ in the CV model can be placed in any position. Furthermore, CV is able to obtain indistinct contours inside or outside the object and shrink or expand simultaneously as shown in Figs. 1(e) and 1(f). All of these capabilities do not work well in the GAC. It can also be observed that CV evolution works like an unsupervised clustering algorithm with two classes $c_{1}$ and $c_{2}$. So adjustable μ, $\lambda_{1}$, and $\lambda_{1}$ are not influential. If all of these constants are set with the same fixed values equal to 1, then Eq. (5) is expressed as

$$\frac{\partial \phi }{\partial t}=\{\mathrm{div}(\frac{\nabla \phi }{\left|\nabla \phi \right|})+2(c_{1}-c_{2})[I-0.5(c_{1}+c_{2})]\}\delta (\phi ).$$ (8)

The key success of CV is actually determined by the intensity threshold mechanism $\left[I-0.5(c_{1}+c_{2})\right]$ that changes ϕ at every point pixel during the evolution. By eliminating $2(c_{1}-c_{2})$ and maintaining the curvature used, similar performance can still be obtained using a simplified formulation, i.e.,

$$\frac{\partial \phi }{\partial t}=\{\mathrm{div}(\frac{\nabla \phi }{\left|\nabla \phi \right|})+[I-0.5(c_{1}+c_{2})]\}\delta (\phi ).$$ (9)

Figs. 1(g) and 1(h) show the US segmentation using simplification CV that yields the same visualization as its original model.

**Fig. 2:**
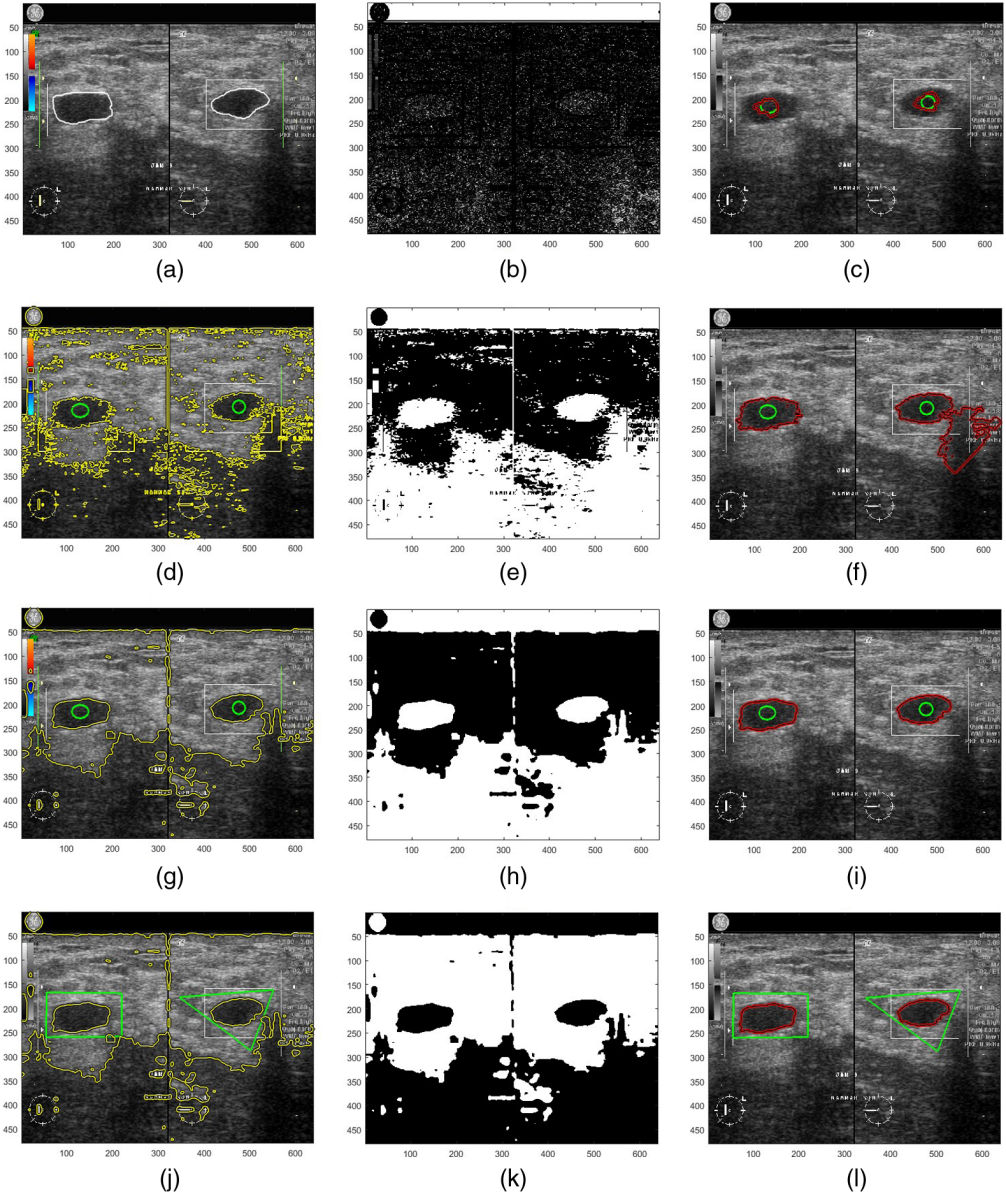
(a) Ground truth. (b) ESF g. (c) GAC using g. (d)–(f) Simplified CV, BSF, and GAC on noisy image. (g)–(i) Simplified CV, BSF, and GAC expanding mode on filtered image. (j)–(l) Simplified CV, BSF, and GAC shrinking mode on filtered image.

Utilizing the advantages of simplified CV evolution, the concept of the BSF is proposed to replace the unreliable ESF g. Referring back to Eqs. (1), (3), and (6), two types of BSF κ can be generated as

$$\kappa \{\begin{array}{ll} H(\phi ) & \text{for expanding}\;\mathrm{GAC},\;\mathrm{i.e.},\;v<0\;\text{and}\;C_{0}\;\text{inside the object}\\ 1-H(\phi ) & \text{for shrinking}\;\mathrm{GAC},\;\mathrm{i.e.},\;v>0\;\text{and}\;C_{0}\;\text{outside the object} \end{array},$$ (10)

where ϕ is the level set function of Eq. (9) after it converges. We can see Fig. 2 for the illustration of both BSF κ. Compared with g, the stopping function κ in the form of a binary image is more robust to providing edge gradient. GAC precisely evolves in the white region and stops in the black region of the related binary image. Unfortunately, the global intensity threshold is only effective for clustering two homogeneous regions. As mentioned in the previous section, noisy US images due to speckle are a major factor in pixel heterogeneity that causes erroneous segmentation, so the mainstay despeckling technique becomes necessary.

### 2.3 Speckle Reduction Bilateral Filter

Based on the investigation of speckle properties by Eom,^32^ it can be transformed in the additive noise model given as

g(i,j)=f(i,j)+h(i,j)*w(i,j), (11)

where g(i,j) is the noised image, f(i,j) is the original input image, and h(i,j) and w(i,j) are the function of point spreading and the Gaussian white noise, respectively. The BF consists of a computational moving window in every image pixel that substitutes the intensity value of the central pixel under the window. A mathematic formulation of the BF is briefly expressed as

$$h(p)=\Gamma^{-1}(p)\int_{\Omega (p)}f(\zeta )s(\zeta ,p)i[f(\zeta ),f(p)]d\zeta ,$$ (12)

where

$$\Gamma (p)=\int_{\Omega (p)}s(\zeta ,p)i[f(\zeta ),f(p)]d\zeta ,$$ (13)

where f and h are the input and output image, respectively. Ω(p) is the neighborhood of spatial coordinate pixel p in the image, and ζ is a variable of integration pixel coordinates in Ω. s(ζ,p) and i[f(ζ),f(p)] are defined as

$$s(\zeta ,p)=\exp(\frac{-{\left\|p-\zeta \right\|}^{2}}{2\sigma_{s}^{2}}),$$ (14)

$$i[f(\zeta ),f(p)]=\exp(-\frac{{\left[f(p)-f(\zeta )\right]}^{2}}{2\sigma_{i}^{2}}),$$ (15)

where $\sigma_{s}$ and $\sigma_{i}$ are the Gaussian standard deviation values on the spatial and range domains, respectively. Equation (14) spatially weights the Euclidean distance between s and ζ, and Eq. (15) operates on the intensity domain.

A speckle reduction BF as preprocessing step is employed here for accurate US segmentation. A BF^26^ is a kind of smoothing low-pass filter that is able to enhance lesion boundaries without destroying important features.^23^ It is a non-linear filter that uses a range filter along with a spatial filter. The segmentation of simplified CV on the BF filtered image produces a better BSF. As shown in Figs. 2(g)–2(l), GAC evolution that applies the BSF from filtered images is kept from leaking and is free from being trapped into local minimal. While GAC that implement the BSF created from noisy images is still prone to leakage such as in Figs. 2(d)–2(f).

### 2.4 Proposed ACBF, Combinatorial Active Contour with Bilateral Filter

In short, the aforementioned descriptions show that US segmentation is obtained through three steps, i.e., image enhancement by the BF, BSF creation using simplified CV, and local object delineation with GAC. To integrate all of these main procedures, a combinatorial hybrid model of an active contour BF called the ACBF is introduced in this paper. Given that the variational Dirac δ(ϕ) works similar to |∇ϕ|,^19^^,^^31^ then the proposed ACBF hybrid model is formulated by combining the simplified CV in Eq. (9) with the GAC model in Eq. (2) as

$$\frac{\partial \phi }{\partial t}=\{\mathrm{div}(\kappa \frac{\nabla \phi }{\left|\nabla \phi \right|})+(1-|\alpha |)[I_{BF}-(\frac{c_{1}+c_{2}}{2})]+\alpha \kappa \}|\nabla \phi |,$$ (16)

where $I_{BF}$ denotes the enhanced US image by the BF. In addition to having the same role as v to determine the deformation mode, α is also a switch control from global into local evolution.

$$\alpha \{\begin{array}{ll} 0 & \text{initial value to start global evolution forcreating}\;\kappa \\ -1 & \text{local evolution for expanding mode}\;\mathrm{i.e.}\;\sum \phi (\nabla C_{0})<0(C_{0}\;\text{inside the object})\\ +1 & \text{local evolution for shrinking mode}\;\mathrm{i.e.}\;\sum \phi (\nabla C_{0})>0(C_{0}\;\text{outside the object}) \end{array}.$$ (17)

**Fig. 3:**
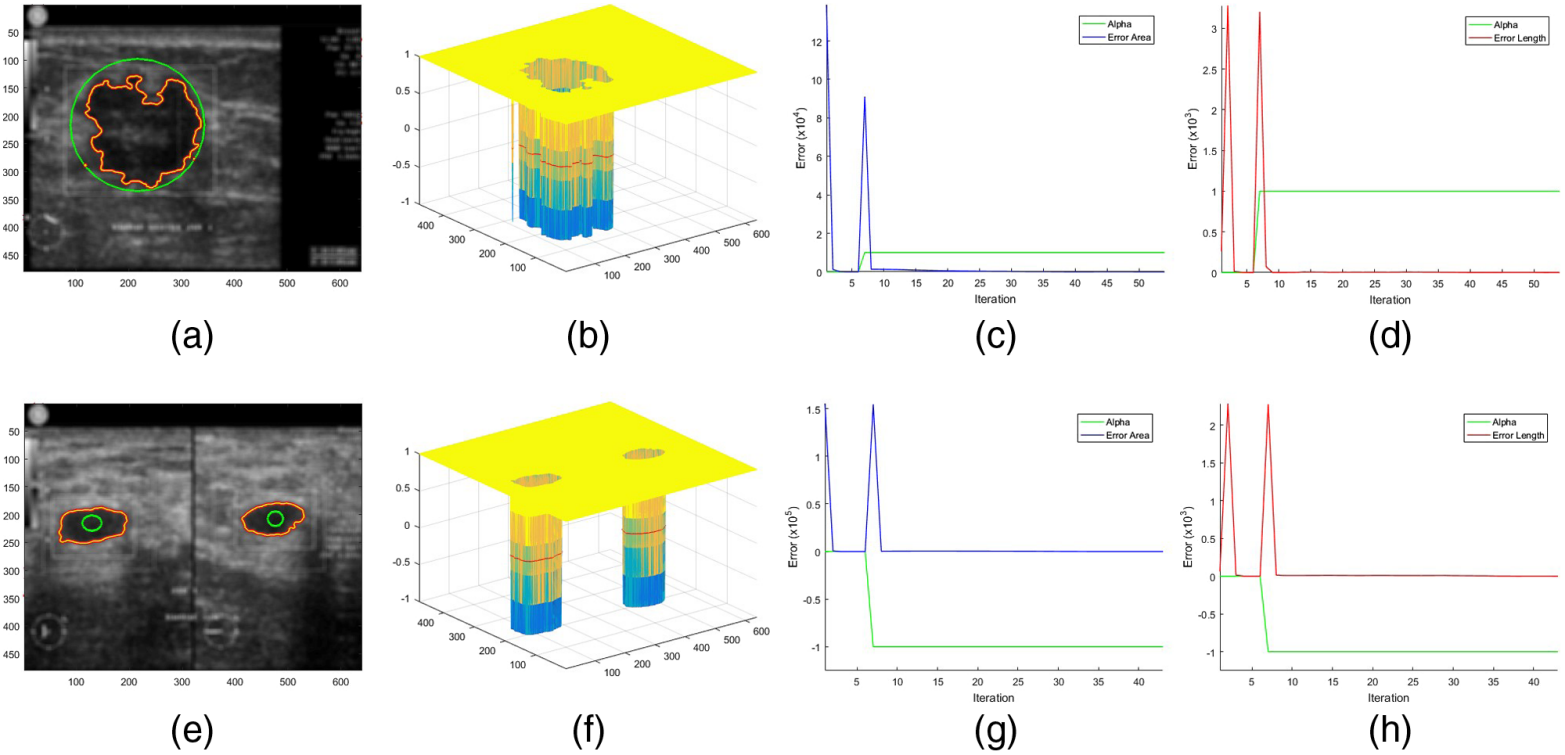
(a) and (e) ACBF shrinking (first row) versus expanding (second row) mode. (b) and (f) Final level set. (c) and (g) Trend of error area. (d) and (h) Error length.

For the initial condition, the values of κ=1 and α=0 are used, considering that BSF can only be created if global evolution has converged. Returning to the base definition in Eq. (1), the sum pixels of inside the zero-level set contour is negative and that outside is positive. Thus, the deformation mode can automatically be determined using the edge pixel location of initial region ($\nabla C_{0}$) in the level set function. Furthermore, the convergence criterion based on tolerable error area and contour length is also established so that the switching mechanism from global (α=0) to local (α=±1) evolution takes place adaptively. Utilizing Eq. (6), the perimeter length and object area are represented as

$$\mathrm{Length}\{\phi =0\}=\int_{\Omega }|\nabla H[\phi (x,y)]|dx\;dy=\int_{\Omega }\delta [\phi (x,y)]|\nabla [\phi (x,y)]|dx\;dy,$$ (18)

$$\text{Area}\{\phi <0\}=\int_{\Omega }H[\phi (x,y)]dx\;dy,$$ (19)

then convergence criterion is expressed as

$$\text{Error Length}=\mathrm{Length}(\phi^{i+1})-\mathrm{Length}(\phi^{i})\leq \theta ,$$ (20)

and

$$\text{Error Area}=\mathrm{Area}(\phi^{i+1})-\mathrm{Area}(\phi^{i})\leq \theta ,$$ (21)

where θ≈0 to specify the small change in tolerable contour deformation. Computational efficiency is indicated by the minimal iteration i to achieve convergence. Trends in error length and area during ACBF evolution are shown in Fig. 3. Adaptive alpha switching is shown by a green line. Two peak error lines indicate a transition from global to local evolution marked by alpha changes from 0 to ±1.

In the traditional level set, the evolution is initialized by the signed distance function to its contour interface to prevent it from being too flat or steep near its contour; then the re-initialization procedure is required in AC evolution. However, many existing reinitialization techniques have an unexpected side effect of moving the AC from its contour interface. Moreover, the re-initialization procedure is an expensive computation.^33^ Here, we adopt an efficient regularization method,^31^ which applies an RD concept to penalize the binary level set function during evolution of the ACBF. The main steps of the proposed ACBF are detailed as follows:

1. Apply the BF to enhance the US images according to Eq. (12). Examples of filtered images are shown in Figs. 3(a) and 3(e).
2. Set values κ=1, α=0 and initial level set $\phi_{0}$ by Eq. (3). More $C_{0}$ can be made for segmenting multiple lesions as shown by the initial green contours in Figs. 2 and 3(e).
3. Evolve the ACBF hybrid formulation in Eq. (16). Illustration of the process and results can be seen in Fig. 3.
4. Let level set ϕ=1 if ϕ>0; otherwise, ϕ=−1. Then do reaction-diffusion regularization ϕ=ϕ+0.2Δϕ, where Δ is the discrete Laplacian operator. Regularized level set functions are depicted in Figs. 3(b) and 3(f).
5. Check convergence criterion by Eqs. (20) and (21). If not yet converged, go back to step 3. Convergent level set evolution is indicated by the both blue and red lines ending straight at score Error=0 in Figs. 3(c) and 3(d) and 3(g) and 3(h).
6. Generate BSF κ in Eq. (10) and switching control α in Eq. (17) adaptively. The BSF is visualized in Figs. 2(e), 2(h), and 2(k) while the change of α is shown by green lines in Figs. 3(c) and 3(d) and 3(g) and 3(h).
7. Return to steps 3 to 5 and stop if it converges.

## 3 Results and Discussion

### 3.1 Comparative Results

**Fig. 4:**
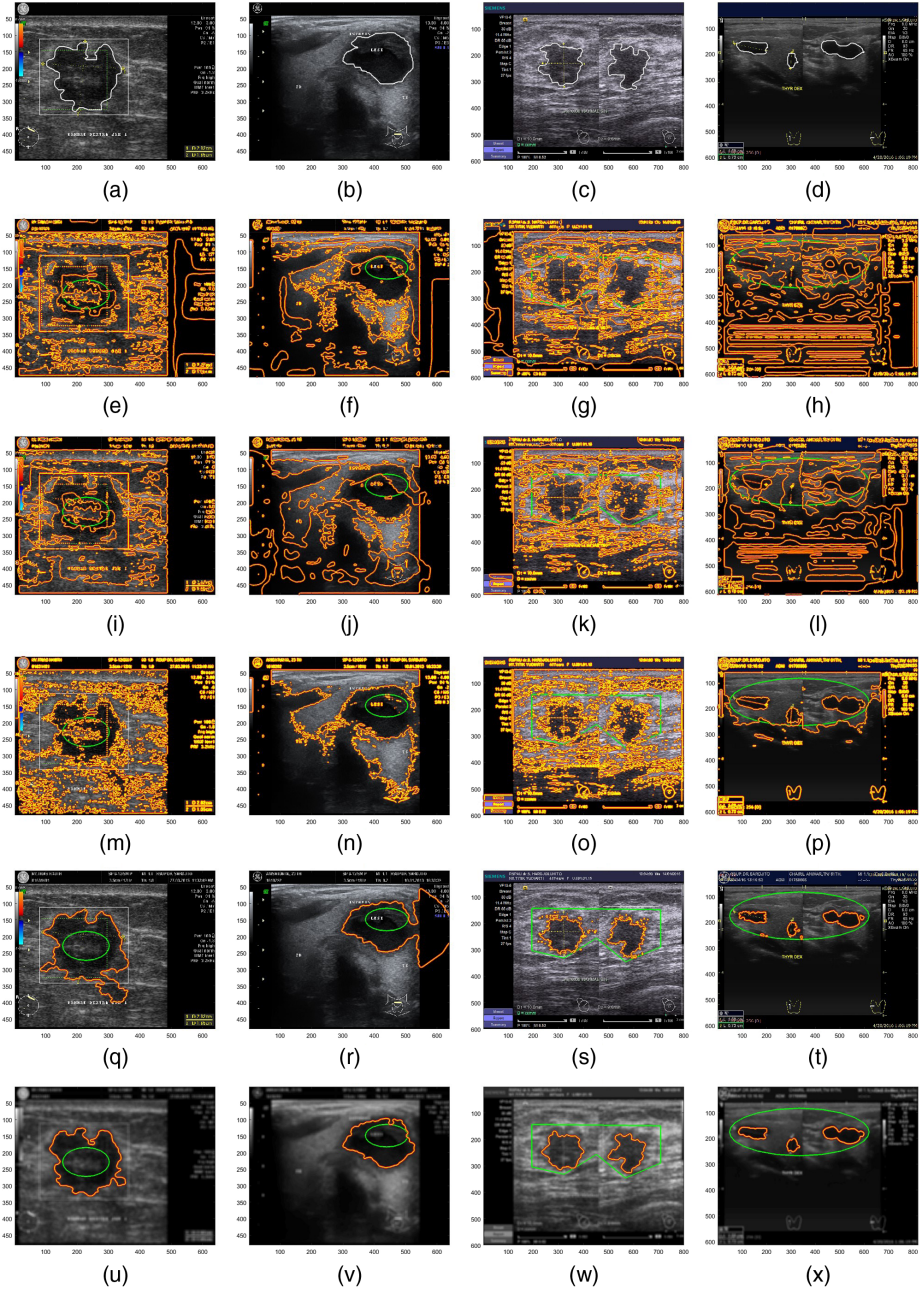
(a)–(d) Ground truth; (e)–(h) LBF; (i)–(l) LGBF; (o)–(p) GACV; (q)–(t) hybrid-AC; and (u)–(x) ACBF.

All of the descriptions above have clearly shown the advantages of the proposed method over CV and GAC. As for demonstrating the performance of the proposed method to the other two, we compare it with the LBF,^33^ hybrid LGBF,^34^ and GACV^14^^,^^21^ models. Segmentation results are visualized for different real US images in Fig. 4. The same level-set initializations and regularization technique^31^ are applied in each method for objective comparison purposes. LBF and LGBF are AC models based on Gaussian localization to overcome image inhomogeneity. However, severe heterogeneity on US images is more effectively solved by a reliable evolutionary stop function. As can be seen in the second and third rows, these two models fail to achieve convergence. Instead of contour localization, these models actually spread like a global segmentation approach. In line with the authors, efforts to combine CV and GAC are also taken on the GACV model. However, the weakness of ESF g which is based on trial and error has become a failure factor for the GACV model in segmenting US images. With the failure of this function, the GACV works just like a global CV segmentation. It can be seen that Fig. 4(m) gives a similar visualization as Figs. 1(e)–1(h). The role of the BSF in the proposed model is able to localize the contour evolution well. Rows 5 and 6 show that the BF is quite effective at improving the BSF to prevent the possibility of leakage and local-minimal traps.

**Fig. 5:**
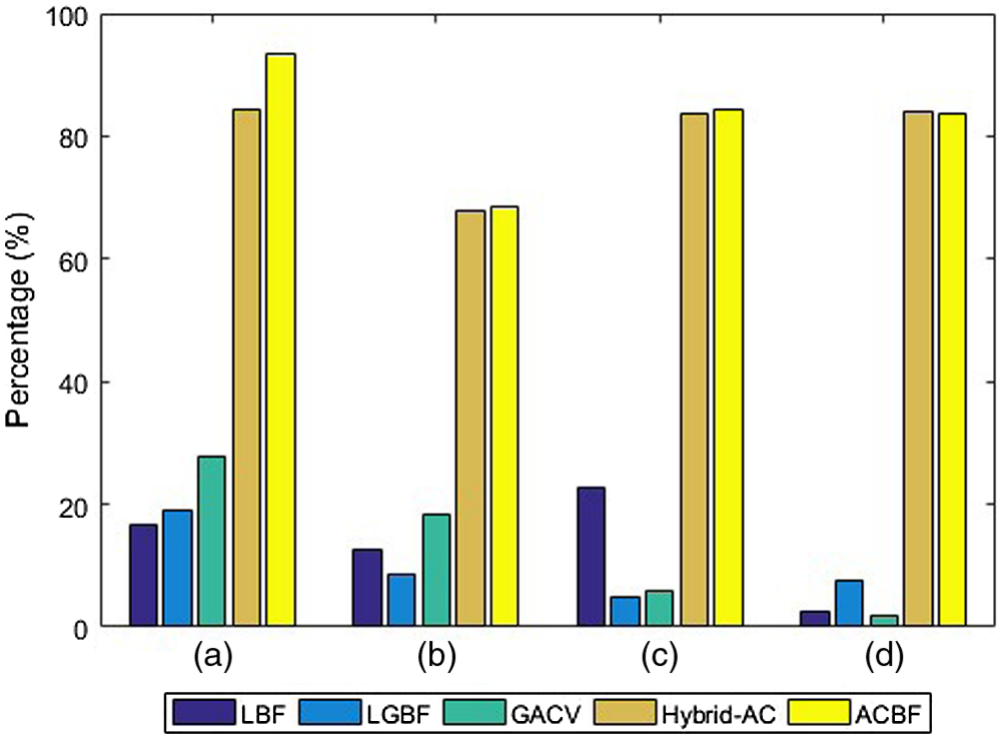
DC values of different methods in Fig. 4.

Furthermore, the performances of these models are measurably assessed by the DC^6^^,^^12^ given as

$$\mathrm{DC}=\frac{2|A_{pm}\cap A_{gt}|}{\left|A_{pm}|+|A_{gt}\right|}x100\%,$$ (22)

where $A_{pm}$ is the area of pixels resulted by proposed method and $A_{gt}$ is the ground truth area. The higher the DC value is, the more accurate the results are. Quantitative measurements in Fig. 5 show that the two proposed models have the highest scores. The computational cost of segmentation in Fig. 4 is reported in Table 1. The proposed models consume the shortest computational time as they are able to achieve convergence while other methods fail. As long as the BSF works steadily, computational costs are determined more by how large the object that will be segmented is. Evaluation on the larger scale image segmentation will provide a more objective analysis.

**Table 1:** CPU computation time (in s) of the images in Fig. 4.

Related methods	Image (a)	Image (b)	Image (c)	Image (d)
LBF	90.16	165.52	476.69	92.78
LGBF	169.36	174.17	237.97	254.39
GACV	29.64	29.36	45.63	45.75
Hybrid-AC	25.41	12.05	14.16	24.98
ACBF	18.23	9.91	14.19	13.06

### 3.2 Performance on a Larger Scale Segmentation

In this study, 258 radiological US images consisting of thyroid nodules and breast lesions were used to validate the performance of the proposed method. All of these images were collected from the Department of Radiology, National Central Hospital Sardjito,^35^ Air Force Central Hospital Hardjolukito Yogyakarta, Indonesia, dataset of Thammasat University and Queen Sirikit Center of Breast Cancer of Thailand, and Ultrasound Department Institute of Fundamental Technological Research Polish Academy of Sciences. Each lesion is clinically outlined by radiologists, and manual segmentation is aided as ground truth for validation. Bland-Altman plot^36^ and statistical boxplot are used to visualize data scatter of DC values among segmentation results.

**Fig. 6:**
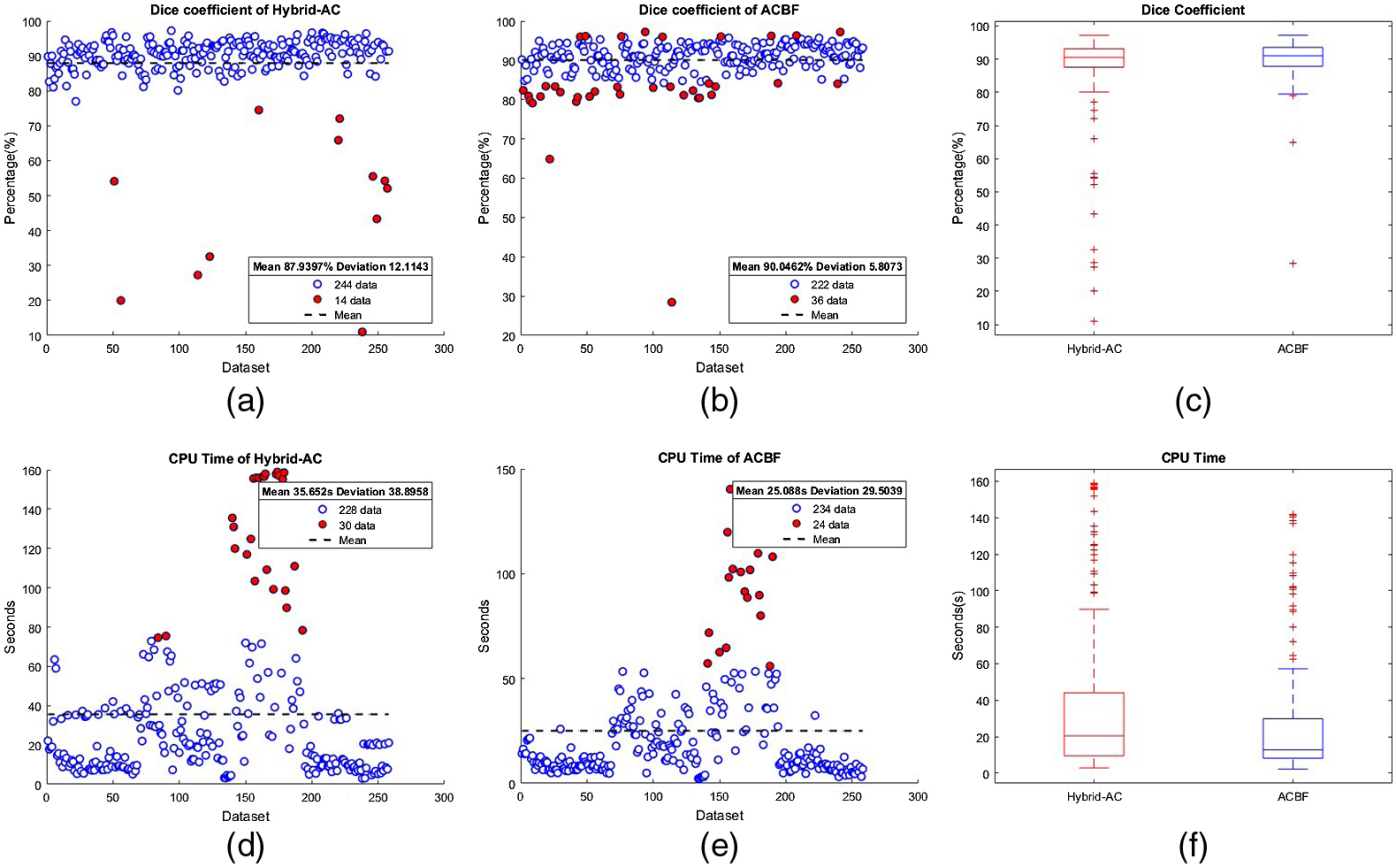
Performance of proposed hybrid-AC model and ACBF in large scale implementation.

From the illustration of Fig. 6, the DC score on the segmentation of US images using the hybrid-AC model is 87.94±12.11%. The role of the BF increased the average performance of DC scores into 90.05±5.81%. Reduction in deviation value implies that the overall DC score is closer to the mean. From the boxplot view, even though it looks the same, the BF provably reduces the outlier data. This shows that the robustness of the BSF in the ACBF is proven. The most important evaluation that needs to be addressed is the computational efficiency of the proposed model. Although the implementation of the BF on hybrid-AC considerably reduces the average CPU time from 35.65±38.90 to 25.09±29.50  s, this value is still quite high for computing costs. Moreover, the high value of the deviation implies that there are many data that are very different from the mean value. This is also shown by the many outliers in the boxplot view. Overall, the proposed method works well. This achievement indicates that the proposed ACBF is feasible to be implemented in a US CAD system.

## 4 Conclusion

In this paper, a combinatorial framework called the ACBF is introduced for accurate segmentation of radiological US images. The framework consists of a speckle reduction BF as preprocessing followed by a hybrid AC model to segment the cancerous object both globally and locally. The major benefits of ACBF is that it can effectively delineate multiple lesions in severe speckle noised images, overcoming erroneous segmentation in other models. Increasing the efficiency in this model is an urgent need to be done. Furthermore, how to make it fully automated is also very challenging future work.
